# Comorbid Patterns in the Homeless Population: A Theoretical Model to Enhance Patient Care

**DOI:** 10.5811/westjem.2021.10.52539

**Published:** 2022-02-23

**Authors:** Kanwalgeet Hans, Luke Mike, Robert Heidel, Paula Benavides, Robert Arnce, Jan Talley

**Affiliations:** * Kansas City University, Department of Emergency Medicine, Kansas City, Missouri; † University of Tennessee, Knoxville, Tennessee

## Abstract

**Introduction:**

From the perspective of social determinants, homelessness perpetuates poor health and creates barriers to effective chronic disease management, necessitating frequent use of emergency department (ED) services. In this study we developed a screening algorithm (checklist) from common comorbidities observed in the homeless population in the United States. The result was a theoretical screening tool (checklist) to aid healthcare workers in the ED, including residents, medical students, and other trainees, to provide more efficacious treatment and referrals for discharge.

**Methods:**

In this retrospective cohort study we used the Nationwide Emergency Department Sample (NEDS) to investigate comorbidities and ED utilization patterns relating to 23 injury-related, psychiatric, and frequent chronic medical conditions in the US adult (≥18 years of age) homeless population. Cases were identified from the NEDS database for 2014–2017 using International Classification of Diseases, 9th and 10 revisions, and Clinical Classification Software diagnosis codes. We performed a two-step cluster analysis including pathologies with ≥10% prevalence in the sample to identify shared comorbidities. We then compared the clusters by sociodemographic and ED-related characteristics, including age, gender, primary payer, and patient disposition from the ED. Chi-square analysis was used to evaluate categorical variables (ie, gender, primary payer, patient disposition from the ED), and analysis of variance for continuous variables (age).

**Results:**

The study included 1,715,777 weighted cases. The two-step cluster analysis identified nine groups denominated by most prevalent disease: 1) healthy; 2) mixed psychiatric; 3) major depressive disorder (MDD); 4) psychosis; 5) addiction; 6) essential hypertension; 7) chronic obstructive pulmonary disease (COPD); 8) infectious disease; and (9) injury. The MDD, COPD, infectious disease, and Injury clusters demonstrated the highest prevalence of co-occurring disease, with the MDD cluster displaying the highest proportion of comorbidities. Although the addiction cluster existed independently, substance use was pervasive in all except the healthy cluster (prevalence 36–100%). We used the extracted screening algorithm to establish a screening tool (checklist) for ED healthcare workers, with physicians as the first point of contact for the initial use of the screening tool.

**Conclusion:**

Healthcare workers in the ED, including residents, medical students, and other trainees, provide services for homeless ED users. Screening tools (checklists) can help coordinate care to improve treatment, referrals, and follow-up care to reduce hospital readmissions. The screening tool may expedite targeted interventions for homeless patients with commonly occurring patterns of disease.

## INTRODUCTION

Homelessness, which we define in this study as self-identified, ongoing problems with access to safe and affordable housing,^1^ is a major societal issue worldwide. The United States Interagency Council on Homelessness released a strategic plan to end homelessness, and defined homelessness as the following: 1) rooflessness; 2) houselessness; 3) living in an insecure accommodation; or 4) living in an inadequate accommodation.^2^ The US recorded an increase of 2.7% in homeless persons from 2018 through 2019, or approximately 570,000 people living without a residence.^3^ The homeless population in the US has been composed of a wide range of people, including single women (40%), families (36%), and unaccompanied adolescents (6.5%).^3^ The World Health Organization has reported that consistent with the US population in general, the US homeless population is living longer and the number of co-existing disorders for individuals has increased, resulting in higher rates of morbidity and premature mortality.^1^

The causes of homelessness are multidimensional and include poverty, unemployment, lack of affordable housing, domestic violence, sexual assault, family breakdown, and adverse childhood experiences.^4^–^8^ O’Neill has recommended that a screening tool for social determinants of health should be used to evaluate risk factors related to health outcomes^9^ because homelessness is also associated with concomitant medical, psychiatric, and addictive disorders.^10^ Traditionally EDs have addressed homeless patients within the context of disease and the episodic need for treatment. More research is needed to determine the prevalence and characteristics of home-less persons treated in EDs to develop evidence-based treatment strategies that address social determinants of health for homeless persons, such as the need for stable and secure housing, access to follow-up care appropriate to the need for care, food, and clean clothing as well as hygienic facilities.^3^,^4^–^6^,^8^–^12^

Social determinants of health affect exposure, onset, access to treatment, and response to communicable diseases. Where the homeless live and are treated affect non-communicable diseases as well.^12^–^17^ Emergency departments need to become part of a continuum of care that begins with acute care and seamlessly transfers to primary care as well as to global healthcare. Clinicians in the ED may benefit from formal education in screening, brief intervention and referral to treatment (SBIRT) training, addiction medicine, the pernicious effects of stigma, and other social determinants of health to increase efficacious treatment for homeless patients who must navigate the healthcare system.^18^

Previous studies using data from the Veterans Health Administration to perform cluster analyses identified sociodemographic characteristics and patterns of psychiatric and general medical comorbidities of homeless veterans to improve care for these patients.^2^–^5^ General recommendations for treatment have also been established for homeless individuals in the primary care setting, although not in the ED, according to the National Health Care for the Homeless Council in 2020.^10^ More research is needed to determine the prevalence and characteristics of homeless persons treated in EDs as well as to develop evidence-based treatment strategies that address social determinants of health for homeless persons,^4^,^5^ such as the need for stable and secure housing, access to appropriate follow-up care, availability of medical respite programs, and hygienic facilities.^2^,^8^–^12^ ^In^ a review of the research published to date we found few studies that described the most common comorbidities seen among homeless persons seeking ED services in the US.

**Population Health Research Capsule**

What do we already know about this issue?

*Adult homelessness, a social determinant of health, contributes to poor health outcomes and ineffective chronic disease management that in turn leads to frequent ED use.*

What was the research question?

*Can a screening tool (checklist) be developed to describe protocols for common comorbidities for homeless adults in EDs in the US?*

What was the major finding of the study?

*Screening algorithms (checklists) were developed using a national ED database to treat adult homeless patients.*

How does this improve population health?

*Clinicians in the ED can use screening tools (checklists) to expedite targeted interventions for adult, homeless patients with commonly co-occurring disorders.*

A screening tool (checklist) for ED healthcare workers, residents and medical students is needed.^19^–^21^ Screening tools (checklists) used in hospital settings have been documented to improve medical performance in the following ways: 1) establishing a higher baseline of performance^22^; 2) improving physician protocols for the unexpected patient response to a procedure^23^; and 3) training physicians to attend to important stimuli by using checklists during complicated clinical encounters to enhance memory and attention, and improve problem solving.^24^ Three types of checklists that have improved healthcare worker performance in medical settings included the following: 1) protocols for normal, routine treatment; 2) protocols for communication; and 3) protocols to manage nuance and unpredictability.^25^,^26^ In addition to improving the efficacy of care delivered in EDs by residents and medical students, data acquired from an ED checklist could serve to improve performance for all healthcare workers.^25^–^27^

Emergency physicians and residents may benefit from formal education in SBIRT training, addiction medicine, the pernicious effects of stigma, and learning the effects of social determinants of health to deepen their understanding of the context in which homeless patients navigate the healthcare system to seek treatment.^5^,^18^ According to the literature, evidence-based curricula focusing on educating residents and other healthcare professionals on effective approaches in caring for homeless ED patients have been lacking.^5^ A prior study, which elaborated on these training deficits with medical residents, reported the use of stereotypical presentations of poverty and lack of cleanliness to identify homeless patients in the ED. As a consequence, substandard delivery of care was found to be associated with the preconceived perception of homeless persons being a difficult population to treat.^5^,^19^ General recommendations for treatment have been established for homeless individuals in the primary care setting but not in the ED, according to the National Health Care for the Homeless Council in 2020.^10^ These protocols, which were developed in primary care settings with sample populations, may not be generalizable to the homeless population.^28^

In this study our goal was to establish a screening tool (checklist) for emergency clinicians and trainees to improve the treatment of homeless patients in the ED. Grouping patients by similar comorbidities, social factors, demographics, and ED utilization may assist by providing a template from the first point of contact with the physician and continuing to discharge and referral for follow-up care to improve the efficacy of treatment protocols for the homeless throughout their care. This retrospective cohort study used the Nationwide Emergency Department Sample (NEDS) from 2014–2017 to investigate multimorbidity and ED utilization patterns seen in the US adult homeless population during that timeframe.^29^ We hypothesized that an analysis of this database using cluster analysis based on primary and secondary diagnoses and patient dispositions would provide information about the more common co-occurring disorders for homeless persons presenting to EDs, and thus contribute to the development of checklists for protocols to improve treatment by reducing errors caused by lack of attention to social determinants of health, as well as stereotyping and misinformation regarding treatment of homeless persons.

## METHODS

### Study Design and Sample

This retrospective cohort study used the NEDS to investigate multimorbidity and ED utilization patterns seen in the US adult homeless population from 2014–2017.^29^ The NEDS belongs to a network of databases formulated by the Healthcare Cost and Utilization Project (HCUP) sponsored by the Agency for Healthcare Research and Quality (AHRQ). The NEDS contains discharge data collected using a stratified, random sample design from 984 EDs in 36 states. It is the largest ED database, providing US national estimates on trends and other healthcare-specific factors seen in the ED setting. We selected cases for adults 18 years of age or older who self-identified as homeless and were treated in an ED. We identified ED visits in the NEDS database using the *International Classification of Diseases, Ninth Revision, Clinical Modification* (ICD-9-CM), *International Classification of Diseases, Tenth Revision, Clinical Modification* (ICD-10-CM), and *Clinical Classifications Software* (CCS) diagnostic codes.^29^

Within the NEDS database, homelessness was defined based on patients’ “yes” or “no” responses to the question of being homeless. Based on the literature review performed for this study, 23 conditions were found to be the most prevalent pathologies and psychiatric disorders treated in the homeless population.^30^–^37^ However, we used only disorders with a prevalence rate above 10%. Nine disorders met this criterion, accounting for 1,715,777 weighted cases. Variables investigated were patient diagnosis, patient demographics (ie, age, gender), payment source (ie, Medicaid, Medicare, private insurance, self-pay, no charge, and other insurance), and disposition from the ED (ie, routine, transfer to short-term hospital, transfer to other hospital, home healthcare, against medical advice, and inpatient admission).^29^ Institutional review determined that this study was exempt from oversight for human participants’ protection due to the de-identified nature of the database.

### Data Analysis

To investigate the conditions with the highest prevalence rates observed in the ED, we used only those disorders with a prevalence rate above 10% to maintain optimal separation and cohesion between and among the various clusters. Cases were entered into a two-step cluster analysis as appropriate for categorical variables for large datasets.^32^ We assessed cluster quality using the silhouette measure of cohesion and separation. We also calculated the cluster sizes and ratio of the largest to the smallest cluster sizes. We reported and inter-preted descriptive and frequency statistics for the characteristics in each cluster. The clusters obtained from the analysis were compared with respect to sociodemographic and ED-related characteristics, including age, gender, primary payer, and patient disposition from the ED. We used chi-square analysis for evaluating categorical variables (gender, primary payer, patient disposition from the ED) and analysis of variance for continuous variables (age). All analyses were performed using SPSS version 26 (IBM Corporation, Armonk, NY), and we assumed statistical significance at an alpha value of 0.05. To illustrate nationally representative results, the discharge-level weight variable, built into the NEDS database, was subsequently used upon completion of the cluster analysis.^29^

### Screening Algorithm

The screening tool used the comorbidity groupings from the two-step cluster analysis to create a stepwise progression through an algorithmic process, with focus placed on maximizing efficiency when screening disease states. To increase the specificity of patient placement within the algorithm, we used only disease states with a 20% prevalence or higher from that respective cluster. We developed disposition recommendations for each cluster using clinical guidelines published by the National Health Care for the Homeless Council.^10^

## RESULTS

### Sample Characteristics

Listed in order from most to least prevalent, the nine conditions examined in this sample were as follows: substance use disorder (SUD; 43.4%); essential hypertension (HTN; 24.8%); major depressive disorder (MDD; 19.7%); psychotic disorders (16.1%); injuries and accidents (not self-inflicted; 16.1%); anxiety disorders (14.2%); bipolar disorder (13.2%); suicidal ideation (12.9%); infectious disease (10.5%); and chronic obstructive pulmonary disease (COPD; 10.3%). A comprehensive list of relevant ICD-9-CM, ICD-10-CM, and CCS diagnosis codes defining each disease category by specific conditions investigated is available upon request in table format. The 10 disorders with prevalence rates equaling 10% or higher used in the cluster analysis are listed in Table 1.

### Cluster Analysis

Two-step cluster analysis yielded a nine-cluster solution with a silhouette measure of 0.47 indicating fair cohesion and separation among the various clusters. Each cluster was named for the most prevalent disease or disease-type within that cluster, with the exceptions being the “healthy” and “mixed psychiatric” clusters. The prevalence of SUD was 36% or greater in all except the healthy cluster. Another common comorbidity found within more than half the clusters was HTN, which was present in all except the healthy, mixed psychiatric, and addictive disorders clusters (Table 2).

### Demographics

We found that most groups for age were found to have averages ranging from 40–49 years. However, cases from the COPD cluster had an average age of 56.5 years (*P* < 0.001) while those within the mixed psychiatric cluster averaged 39.2 years (*P* < 0.001). The HTN cluster also correlated with an aged patient demographic, with an average of 53.1 years (*P* < 0.001). In terms of patient gender, males predominated, constituting 73% of this sample of ED visits, and nearly 65% (*P* < 0.001) of each cluster, with the exceptions of the mixed psychiatric and addictive disorders clusters exhibiting the high and low ends of this spectrum (proportion of females, 34.6% and 19.6%, respectively; *P* < 0.001). The infectious disease cluster contained the second lowest proportion of females at 22.4% (*P* < 0.001; Table 3).

### Primary Payer

Overall, the primary insurance used by homeless patients in this sample was Medicaid, representing the primary payer-type of nearly 50% of cases (*P* < 0.001) from each cluster. This was especially true for the addictive disorders and injury and accidents clusters (Medicaid coverage for associated charges, 56.0% and 57.5%, respectively; *P* < 0.001). Medicare coverage only accounted for 11.6% of cases (*P* < 0.001), with the psychotic disorders and COPD clusters seeing higher Medicare utilization rates of 27.7% and 32.6%, respectively (*P* < 0.001). Interestingly, the healthy and addictive disorders clusters saw a higher percentage of self-paying patients (21.2% and 21.7%, respectively; *P* < 0.001; Table 3).

### Disposition from the Emergency Department

The patient disposition-type from the ED demonstrated a large variance between clusters. As expected, the healthy cluster showed the highest rate of receiving routine care (79.8%, *P* < 0.001) and being discharged from the ED against medical advice (3.4%, *P* < 0.001). This cluster also showed the lowest rate of inpatient hospital admission (15.1%, *P* < 0.001). Although the addiction, HTN, and injury and accident clusters likewise showed high percentages of routine care (57.8%, 56.8%, 56.1%, respectively; *P* < 0.001), these clusters were also associated with greater percentages of hospital admissions compared to the healthy group (37.1%, 38.0%, and 39.0%, respectively; *P* < 0.001). The highest rate of hospital admission was noted in the infectious disease group, with an admittance rate of 62.4% (*P* < 0.001). Similarly, the COPD and MDD clusters also showed high rates of hospital admission (56.9% and 56.6%, respectively; *P* < 0.001), although the MDD cluster showed the lowest proportion of cases receiving routine care (33.9%, *P* < 0.001). The mixed psychiatric cluster included the youngest cases (39.2 years, *P* < 0.001), the highest proportion of female patients (34.6%, *P* < 0.001), and showed the highest percentage of transfers to another facility (9.4%, *P* < 0.001; Table 4).

### Emergency Department Screening Algorithm

**Figure 1:**
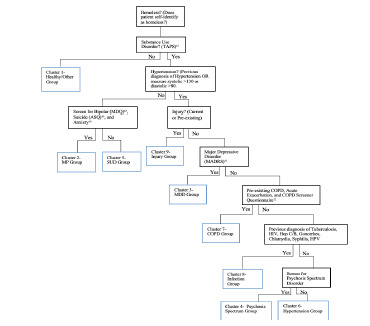
Emergency department medical and psychiatric screening algorithm for homeless individuals. *TAPS,* tobacco, alcohol, prescription medication, and other substance use tool; *MDQ,* mood disorder questionnaire; *ASQ,* ages and stages questionnaire; *MP,* Mixed Psychiatric group; *SUD,* substance use disorder; *MADRS,* Montgomery-Asberg depression rating scale; *MDD,* major depressive disorder; *COPD,* chronic obstructive pulmonary disease; *HIV,* human immunodeficiency virus; *Hep C/B,* hepatitis C and B; *HPV,* human papilloma virus.

The screening algorithm yielded a maximum of eight steps beginning with a positive screen for homelessness. Disease states commonly seen within a cluster were screened for first, and less common disorders were screened in subsequent steps, thereby ensuring that each disease state was screened for only once. This stepwise progression used recommendations for each cluster providing post-discharge treatment options focusing primarily on referrals to primary care and medical specialists capable of delivering definitive treatment (Figures 1 and 2).^38^

## DISCUSSION

Identification of novel strategies to mitigate ED utilization has been a central objective among policy makers in the debate over healthcare reform.^32^–^35^ Evidence suggests that homeless individuals endure a high disease burden and seek ED services at an increased frequency due to a lack of access to chronic disease management services.^21^,^33^–^35^ With the expansion of Medicaid enrollment under the Affordable Care Act, ED interventions identifying comorbidities within the homeless population to provide effective treatment and follow-up care may contain healthcare costs, and improve patient outcomes. The AHRQ has supported several initiatives to enhance the quality of healthcare for people of all social demographics by introducing resources and training, including culturally appropriate methods for communicating with culturally diverse patients in the primary care setting.^31^

In this study we used a two-step cluster analysis method with the NEDS database to examine co-occurring chronic medical, injury-related, and psychopathologies within the adult US homeless population who received ED services from 2014–2017. The resulting nine-cluster solution identified groups of homeless individuals sharing specific demographic characteristics and possessing comorbidities with distinct patterns of ED utilization, possibly contributing important information about the health trends specific to the ED setting and the adult homeless population.

Homeless individuals have been at greater risk for negative health outcomes than the general population, and have exhibited high rates of unintentional injury, psychiatric disorders, substance use, and infectious and chronic diseases.^31^–^34^ The MDD, COPD, infectious disease, and injury and accidents clusters together constituted 45% of all examined cases. These clusters also exhibited the highest prevalence of co-occurring disorders with the MDD cluster having the greatest proportion of comorbidities. While cases in the addiction cluster existed independently from other medical diagnoses, along with psychiatric disorders and HTN, SUD was pervasive among most clusters, with its prevalence ranging from 36–100% in all except the healthy cluster. Substance use disorder was the most commonly diagnosed chronic condition within this study sample.

This study has described potential sociodemographic and ED utilization characteristics of homeless individuals that have been associated with increased hospital costs.^11^,^12^,^35^–^38^ For instance, six of the nine clusters (addiction, infectious disease, HTN, injury and accidents, COPD, and MDD) revealed high rates of hospital admissions, underscoring the need for efficient and effective treatment strategies that can focus on mitigating hospital admission rates. Identification of homeless individuals based on their cluster designations may expedite interventions to ensure treatment that is properly coordinated before discharge. Critical time interventions were found to be effective in mitigating ED utilization by homeless individuals using strategies for enhancing quality of life, securing stable housing, obtaining public income assistance, arranging follow-up primary and specialty care, and increasing accessibility to mental health and substance use treatment.^39^,^40^ These models facilitating continuity of care were also correlated with reduced costs incurred by overburdened hospital systems caring for frequent homeless ED users and those diagnosed with psychiatric disorders.^35^–^37^

The screening tool (checklist) as an algorithm might also provide a template for electronic applications (ie, electronic health records [EHR], smart devices) for EDs, specifically to streamline post-discharge planning for vulnerable homeless patients. EDs have used EHR to provide the following: 1) needed documentation such as referral letters for same-day, primary care appointments; 2) patient records for follow-up care; 3) notifications to prevent the termination of needed services such as housing to prevent eviction notices; and 4) vouchers for travel.^28^

Emergency department-based models that enable real-time collaboration among emergency clinicians and case managers may address some of the unmet healthcare and psychosocial needs of these patients. In-person follow-up by case management after ED discharge was associated with greater costs than telephone-based outreach; however, face-to-face engagement with patients has been associated with better adherence for initial outpatient mental health appointments.^41^

Because addictive disorders were the most prevalent diagnoses found in this study sample, ED treatment protocols need to be augmented with resources and strategies to improve outcomes for addictive disorders, specifically for homeless persons. One evidence-based intervention used by emergency physicians, the SBIRT tool, has been effective at treating both alcoholics and addicts in the ED setting.^1^,^18^ The use of the SBIRT tool to provide referrals for substance use treatment services for a large group of diversified patients resulted in reduced expression of criminal behavior, ameliorated health status, acquisition of safe and affordable housing, and employment. Participants reported a significant reduction in recreational drug use and excessive alcohol consumption with perceived betterment in general and mental health states.^18^

The use of checklists for treatment protocols has been studied and found to increase the use of evidence-based practices for homeless individuals based on cluster designation. Various clinical settings, such as surgery, have used checklists extensively in protocol development and have exhibited improved patient outcomes.^21^–^27^ While recent research has explored the use of electronic checklists, the application of checklists in the ED setting to promote better patient care needs to be explored.^28^ This study’s screening algorithm and associated checklist may also expedite post-ED treatment planning and efficiency by providing necessary medical and psychiatric referrals. Although the proposed screening tool is entirely theoretical because reliability in the ED setting has not been tested, the tool is offered as a template for future research to establish the potential for increased efficacy and efficiency.

While this study described clusters of homeless individuals sharing common comorbidities, addressing the social determinants of health will require action by many community stakeholders including local, state, and federal agencies for funding as well as policy and procedural oversight of services for the homeless. The use of patient-centered medical teams that foster real-time collaboration among all healthcare professionals who work with a patient has been found to contribute to improved health outcomes for that patient.^42^ Patient-centered treatment teams have been established but have had limited application in low-income areas, decreasing the likelihood of providing effective treatment for homeless individuals.^42^

The federal ARHQ report for 2018 stressed the need to examine health-promotion and disease-prevention efforts within the context of populations that are at greater risk as a means to modify and improve exposure to positive social determinants of health. The report stressed the need to examine health promotion and disease prevention efforts not because demographics such as race/ethnicity are genetic but to modify and improve exposure to positive social determinants of health.^31^

## LIMITATIONS

This study had several limitations, especially in relation to the use of the HCUP NEDS database. First, the HCUP did not use a working definition of homelessness; instead it depended on the reporting of partner hospitals for categorizing homeless patients. While this strategy simplified the identification of homeless persons seeking care in the ED, certain state-specific guidelines hindered full reporting practices. As an example, the state of New York did not record non-US born homeless individuals treated in the ED, but rather listed these patients as missing values in their data provided to the HCUP.^43^ Such reporting practices prevented an accurate representation of the actual healthcare and ED utilization trends exhibited by homeless individuals in the US.

Second, in defining case-specific characteristics represented by each cluster, the NEDS database did not record the race of each patient. The National Institute of Human Genome Research has defined race as a fluid dimension that is better understood as ancestral background and/or social identity. Geneticists have proposed that race would be more accurately described as a social and not a biological construct.^44^–^46^ The use of race as a social determinant of health rather than as a genetic construct may contribute to more effective treatment.^44^ Even so, the unfair burden of disease by minority populations as well as disparities in the provision of healthcare must be addressed.^44^–^46^ The medical community must revise medical school curricula as well as treatment protocols by using an equity lens to evaluate medical practice to improve the quality of care for all persons.^9^

Moreover, the NEDS database provides visit-level analysis without identifying individual patients and considers recurrent hospitalizations as distinct cases. With each NEDS entry not being equivalent to one ED admission, there is a potential for one patient to account for multiple entries. While this type of data acquisition exemplifies true adherence to the Health Insurance Portability and Accountability Act privacy rules, it can result in the overestimation of certain diagnoses within the homeless population, with multiple cases associated with the same patient being subject to different outcomes. Another factor complicating the identification of disease patterns in the US homeless population has been variation in the interpretation of medical records by reviewers entering ICD-9 and ICD-10 codes into the EHR. Lack of rater training and retraining may increase the potential for error.

Lastly, because this study used the NEDS database to examine over 1.715 million weighted (or over 390,000 unweighted) cases, very small differences in sociodemographic and ED utilization variables between clusters produced statistically significant differences, making interpretation of valid findings based solely on *P-*values potentially less clinically relevant.

## CONCLUSION

Through a two-step cluster analysis, this nationally representative sample of US ED visits identified groups of homeless patients based on discrete comorbid and ED utilization patterns. Significant comorbidity was found in each of the nine groups especially within the major depressive disorder, COPD, infectious disease, and injury and accidents clusters. Most notably, substance use disorder was ubiquitous among the cases examined, making it the most prevalent chronic condition within our study sample. The proposed screening algorithm may provide a collaborative approach to comprehensive, ED health planning focused on the equitable delivery of high-quality care to all homeless individuals while mitigating repeat hospital admissions.

## Supplementary Information



## Figures and Tables

**Table 3:**
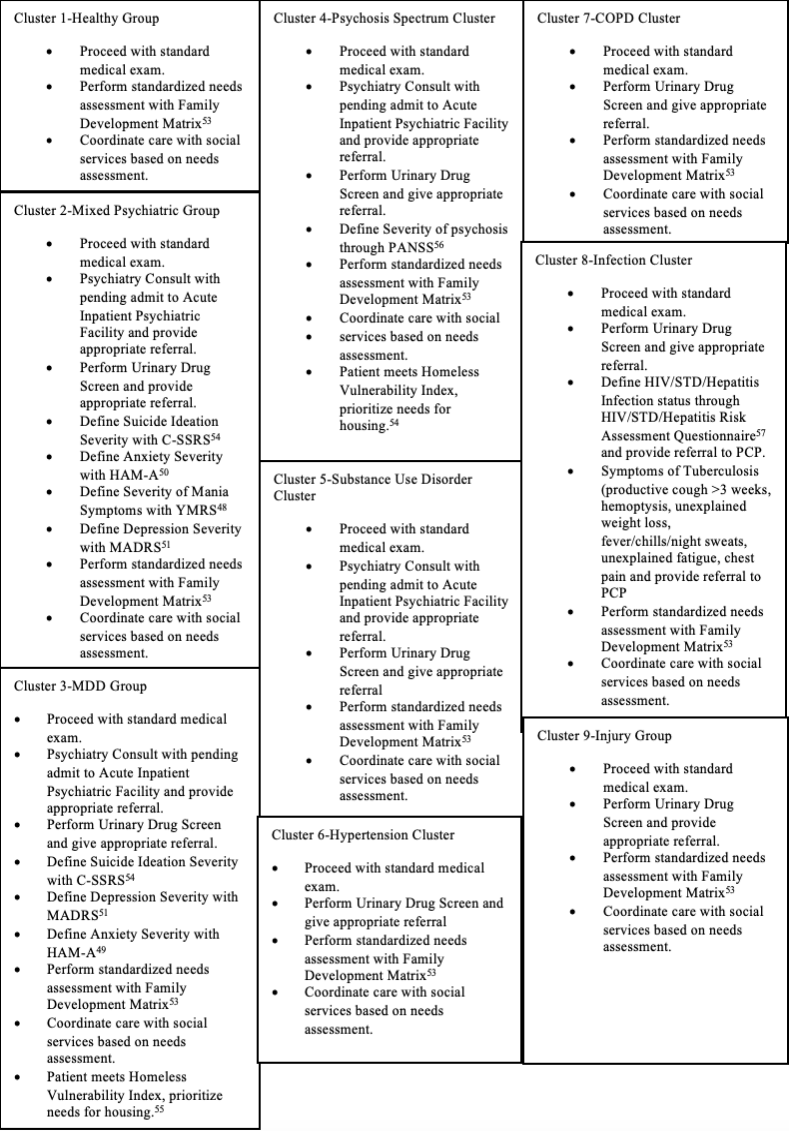
Demographics, primary payer, associations with distibutions C-SSRS, Columbia-Suicide Severity Rating Scale; HAM-A; Hamilton Anxiety Rating Scale; YRMS, Young Mania Rating Scale; HIV, human immunodeficiency virus; STD, sexually transmitted disease; PCP, primary care physician.

**Table 1:** Sample demographics and disease prevalence of the homeless emergency department cases.

	Frequency, n (%)
Sex	
Male	1,251,869 (73)
Diagnosis	
Hypertension	426,302 (24.8)
Psychosis	275,796 (16.1)
COPD	176,123 (10.3)
Anxiety	244,298 (14.2)
Bipolar	226,987 (13.2)
MDD	337,199 (19.7)
Addictive Disorders	744,261 (43.4)
Injuries and Accidents	276,282 (16.1)
Infectious Diseases	180,567 (10.5)
Suicide	221,156 (12.6)

*COPD*, chronic obstructive pulmonary disease; *MDD*, major depressive disorder.

**Table 2:** Two-step cluster analysis of psychiatric, injury-related, and medical conditions with a prevalence of 10% or higher in the emergency department.

Cluster	Sampled weighted ED visits n (%)	SI n (%)	INJ n (%)	HTN n (%)	COPD n (%)	MDD n (%)	BP n (%)	Psychosis, n(%)	ANX n (%)	Infectious n (%)	Addictive n (%)
1	315,373 (18.4)	0 (0.0)	0 (0.0)	0 (0.0)	0 (0.0)	0 (0.0)	0 (0.0)	0 (0.0)	0 (0.0)	360 (0.1)	0 (0.0)
2	156,295 (9.1)	73,337 (46.9)	3,724 (2.4)	0 (0.0)	516 (0.3)	0 (0.0)	77,197 (49.4)	23,007 (14.7)	61,181 (39.1)	4,157 (2.7)	79,031 (50.6)
3	337,199 (19.7)	112,973 (33.5)	46,404 (13.8)	97,468 (28.8)	36,156 (10.7)	337,199 (100.0)	47,333 (14.0)	54,424 (16.1)	110,222 (32.7)	43,628 (12.9)	194,650 (57.7)
4	145,522 (8.5)	5,595 (3.8)	0 (0.0)	33,849 (23.3)	0 (0.0)	0 (0.0)	27,553 (18.9)	145,522 (100.0)	14,431 (9.9)	74 (0.1)	54,491 (37.4)
5	155,064 (9.0)	0 (0.0)	0 (0.0)	0 (0.0)	0 (0.0)	0 (0.0)	0 (0.0)	0 (0.0)	0 (0.0)	107 (0.1)	155,064 (100.0)
6	169,495 (9.9)	10,582 (6.2)	0 (0.0)	169,495 (100.0	0 (0.0)	0 (0.0)	20,727 (12.2)	0 (0.0)	180,900 (10.6)	100 (0.1)	62,469 (36.9)
7	115,691 (6.7)	6,423 (5.6)	937 (0.8)	50,138 (43.3)	115,691 (100.0)	0 (0.0)	16,460 (14.2)	14,522 (12.6)	13,273 (11.9)	14,875 (12.9)	45,416 (39.3)
8	95,922 (5.6)	5,608 (5.8)	0 (0.0)	25,531 (26.6)	25,531 (26.6)	0 (0.0)	14,076 (14.7)	15,411 (16.1)	9,035 (9.4)	95,922 (100.0)	59,514 (62.0)
9	22,216 (13.1)	6,639 (2.9)	225,216 (100.0)	49,821 (22.1)	17,769 (7.9)	0 (0.0)	23,640 (10.5)	22,910 (10.2)	17,707 (7.9)	21,668 (9.6)	93,624 (41.6)

*SI*, suicidal ideation; *INJ*, injury; *HTN*, hypertension; *COPD*, chronic obstructive pulmonary disease; *MDD*, major depressive disorder; *BP*, bipolar affective disorder; *ANX*, anxiety.

**Table 3:** Demographics, primary payer, associations with distributions among nine clusters.

Variable	Healthy, n (%)	Mixed Psychiatric, n (%)	MDD, n (%)	Psychotic, n (%)	Addictive, n (%)	HTN, n (%)	COPD, n (%)	Infectious, n (%)	Injury and Accident, n (%)
Age at admission	46.0 (14.2)	39.2 (12.4)	43.9 (13.1)	42.5 (13.0)	44.6 (12.9)	53.1 (11.2)	56.5 (9.7)	48.0 (11.6)	46.6 (13.5)
Gender (female)	90,051 (28.6)	54,088 (34.6)	103,632 (30.7)	39,316 (19.6)	30,428 (19.6)	41,428 (24.7)	31,709 (27.4)	21,492 (22.4)	51,169 (22.7)
Primary Payer									
Medicare	58,764 (18.7)	29,164 (18.7)	62,055 (18.4)	40,320 (27.7)	17,915 (11.6)	41,104 (24.3)	37,668 (32.6)	17,003 (17.8)	40,055 (17.8)
Medicaid	158,977 (50.5)	80,800 (51.8)	174,007 (51.7)	69,043 (47.5)	86,851 (56.0)	82,683 (48.8)	55,274 (47.8)	55,020 (57.5)	114,910 (51.1)
Private	16,916 (5.4)	12,068 (7.7)	26,209 (7.8)	7,363 (5.1)	8,374 (5.4)	8,923 (5.3)	4,846 (4.2)	4,596 (4.8)	13,366 (5.9)
Self-pay	66,755 (21.2)	27,107 (174)	55,807 (16.6)	23,440 (16.1)	33,557 (21.7)	27,917 (16.5)	12,641 (10.9)	15,555 (15.2)	44,792 (19.9)
No charge	4,966 (1.6)	2,177 (1.4)	6,649 (2.0)	1,494 (1.0)	3,310 (2.1)	3,101 (1.8)	1,472 (1.3)	1,573 (1.6)	3,611 (1.6)
Other	8,476 (2.7)	4,658 (3.0)	11,735 (3.5)	3,702 (2.5)	4,997 (3.2)	5,541 (3.3)	3,660 (3.2)	3,000 (3.1)	8,086 (3.6)

All analyses were statistically significant with a *P*-value less than 0.001.

*MDD,* major depressive disorder; *HTN,* hypertension; *COPD,* chronic obstructive pulmonary disease.

**Table 4:** Disposition from emergency department associations with distributions among nine clusters.

Variable	Healthy, n (%)	Mixed Psychiatric, n (%)	MDD, n (%)	Psychotic, n (%)	Addictive, n (%)	HTN, n (%)	COPD, n (%)	Infectious, n (%)	Injury and Accident, n (%)
Disposition from ED									
Routine	215,146 (79.8)	69,595 (44.6)	114,211 (33.9)	69,704 (48.0)	89,416 (57.8)	96,111 (56.8)	44,762 (38.8)	32,545 (34.0)	126,078 (56.1)
Transfer to short-term hospital	3,183 (1.0)	14,700 (9.4)	23,473 (7.0)	10,928 (7.1)	2,409 (1.6)	3,622 (2.1)	1,783 (1.5)	1,349 (1.4)	4,451 (2.0)
Transfer to other hospital	3,183 (1.0)	14,700 (9.4)	23,473 (7.0)	10,928 (7.1)	2,409 (1.6)	3,622 (2.1)	1,783 (1.5)	1,349 (1.4)	4,451 (2.0)
HHC	259 (0.1)	169 (0.1)	334 (0.1)	145 (0.1)	90 (0.1)	156 (0.1)	118 (0.1)	60 (0.1)	166 (0.1)
AMA	10,826 (3.4)	2,378 (1.5)	3,188 (0.9)	2,592 (1.8)	4,336 (2.8)	3,752 (2.2)	2,442 (2.1)	1,669 (1.7)	4,322 (1.9)
Admitted to inpatient at current hospital	47,393 (15.1)	66,759 (42.8)	190,419 (56.6)	59,677 (41.1)	57,406 (37.1)	64,302 (38.0)	65,740 (56.9)	58,742 (62.4)	87,774 (39.0)

All analyses were statistically significant with a P-value less than 0.001.

*MDD,* major depressive disorder; *HTN,* hypertension; *COPD,* chronic obstructive pulmonary disease; *HHC,* home healthcare; *AMA,* against medical advice.
