# Supplementary material for: Comorbid Patterns in the Homeless Population: A Theoretical Model to Enhance Patient Care
Source: West J Emerg Med. 2022 Feb 23;23(2):200–10. doi: 10.5811/westjem.2021.10.52539 (PMC8967446; doi:10.5811/westjem.2021.10.52539)
Supplement: Supplementary file 1 [file wjem-23-200-s001.docx]

**Supplementary Table 1**-Prevalence of common diseases in homeless & their corresponding ICD9/ICD10 codes.

| Condition | Prevalence n, (%) | ICD-9, ICD-10, CCS Codes |
| --- | --- | --- |
| Infectious Disease (tuberculosis, HIV, Hepatitis C, Hepatitis B, Gonorrhea, Chlamydia, Syphilis, HPV) | 180,557 (10.52) | 010.x-011.x, 042.x, 070.0, 079.x, 091.x-099.x, 795.x, 796.x  A51.x-A54.x, A52.01, A55.x, A70.x, A71.x, A74.x, B18.x, B20.x  1, 5 |
| Neurological Disorder (Traumatic Brain Injury, Cognitive impairment, Intellectual Disability, Seizure Disorder, Epilepsy) | 144574 (8.42) | 317.x-319.x, 331.83, 345.x, 780.x, 854.0  G31.x, F70.x-F73.x, F78.x-F79.x, S06.x  83, 233 |
| Injuries and accidents (not self-inflicted) | 276282 (16.10) | 249.x-250.x, 272.2, 303.x-305.x, 401.x, 410.x-414.x, 437.x, 459.x, 521.x-523.x, 681.x-682.x, 684.x    E11.x, E78.x, E81.4, E88.x, F10.x-F16.x, I10.x-I11.x, I20.x-I25.x, I87.x, K02.x-K05.x, K09.x, K21.x, L01.x, L03.x, W00.x-W01.x, W03.x-W08.x, W10.x, W13.x-W15.x, W17.x-W19.x, V03.x-V04.x    225-232, 234-236, 239-240, 243-244, 2601-2604, 2609, 2611-2614 |
| Dermatologic conditions (Ulceration, Infection, Cellulitis, Venous Stasis) | 125799 (7.33) | 459.x, 681.x-682.x, 684.x,    I87.x, L01.x, L03.x  197-200 |
| Cancer (Hepatic, Oropharyngeal, Colorectal, Lung/bronchial, Ovarian, Cervical, Breast) | 16762 (0.97) | 153.x-155.x, 161.x-162.x, 180.x, 183.x, 174.x    C10.x, C18.x, C22.x, C20.x, C34.x, C50.x, C53.x, C56.x  14-15, 16, 19, 24, 26-27 |
| Essential Hypertension | 426302 (24.84) | 401.x, 437.x, 459.x  I10.x, I11.x, I87.x  98 |
| Dyslipidemia | 118576 (6.91) | 272.x  E78.x  53 |
| Diabetes mellitus (DM) | 134328 (7.82) | 249.x, 250.x  E11.x  49 |
| Dental/oral pathology | 21023 (1.22) | 521.x-523.x  K02x-K05.x, K09.x, K21.x  136-137 |
| Nutritional deficiencies | 58035 (3.38) | 261.x-269.x, 799.4  V12.1  52 |
| chronic kidney disease (CKD) | 57807 (3.30) | 585.x, 792.5  V42.0, V45.1, V451.1, V451.2, V56.0, V56.1, V56.2, V563.1, V563.2, V56.8  158 |
| Ischemic heart disease (IHD) | 110479 (6.40) | 410.x-414.x  I20.x-I22.x, I24.x-I25.x  100-101 |
| Congestive heart failure (CHF) | 87887 (5.12) | 398.91, 428.x    I09.81, I50.x  108 |
| Chronic obstructive pulmonary disease (COPD) | 176123 (10.26) | 490.x-494.x  J40.x-J45.x, J47.x  127 |
| Anxiety disorders | 244298 (14.24) | 300.x    F41.x  651 |
| Bipolar disorder | 226987 (13.23) | 296.x  F31.x- F33.x |
| Major Depressive Disorder (MDD) | 337199 (19.65) | 296.2-269.3  F32.x- F33.x |
| Substance Use Disorder (SUD) | 744261 (43.38) | 303.x-305.x  F10.x-F16.x  660-661 |
| Post-traumatic stress disorder (PTSD) | 19334 (1.13) | 309.81  F43.x |
| Suicidal Ideation | 221156 (12.89) | E950.x-E959.x  E958.9 |
| Psychotic Disorders | 275796 (16.07) | 293.8, 295.x, 297.x, 298.x  F20.x, F31.x  659 |

**Supplementary Figure 1.** Emergency Department medical and psychiatric screening algorithm for homeless individuals with screening tools and recommendations for medical and social services based on cluster.

Homeless? (Does patient self-identify as homeless?)

Substance Use Disorder? (TAPS)^1^

No

Yes

Cluster 1-Healthy/Other Group

Hypertension? (Previous diagnosis of Hypertension OR measure systolic >130 or diastolic >80.

No

Yes

Injury? (Current or Pre-existing)

Screen for Bipolar (MDQ), Suicide (ASQ)^3^, and Anxiety (GAD-7).

No

Yes

Yes

No

Major Depressive Disorder? (PHQ-9)

Cluster 9-Injury Group

Cluster 2-Bipolar Group

Cluster 5-Substance Use Disorder

Yes

No

Cluster 3-MDD Group

Pre-existing COPD, Acute Exacerbation, and COPD Screener Questionnaire^4^

No

Yes

Cluster 7-COPD Group

Previous diagnosis of Tuberculosis, HIV, Hep C/B, Gonorrhea, Chlamydia, Syphilis, HPV

No

Yes

Cluster 8-Infection Cluster

Screen for Psychosis Spectrum Disorder

No

Yes

Cluster 6-Hypertension Group

Cluster 4-Psychosis Spectrum Cluster

**Supplementary Figure 1 Cont.** Post-Algorithm Recommendations

Cluster 3-MDD Group

- Proceed with standard medical exam.
- Psychiatry Consult with pending admit to Acute Inpatient Psychiatric Facility and provide appropriate referral.
- Perform Urinary Drug Screen and give appropriate referral.
- Define Suicide Ideation Severity with C-SSRS^5^
- Define Depression Severity with MADRS^6^
- Define Anxiety Severity with HAM-A^6^
- Perform standardized needs assessment with Family Development Matrix^2^
- Coordinate care with social services based on needs assessment.
- Patient meets Homeless Vulnerability Index, prioritize needs for housing.^7^

Cluster 1-Healthy Group

- Proceed with standard medical exam.
- Perform standardized needs assessment with Family Development Matrix^2^
- Coordinate care with social services based on needs assessment.

Cluster 2-Bipolar Group

- Proceed with standard medical exam.
- Psychiatry Consult with pending admit to Acute Inpatient Psychiatric Facility and provide appropriate referral.
- Perform Urinary Drug Screen and provide appropriate referral.
- Define Suicide Ideation Severity with C-SSRS^5^
- Define Anxiety Severity with HAM-A^6^
- Define Severity of Mania Symptoms with YMRS^6^
- Define Depression Severity with MADRS^6^
- Perform standardized needs assessment with Family Development Matrix^2^
- Coordinate care with social services based on needs assessment.

Cluster 4-Psychosis Spectrum Cluster

- Proceed with standard medical exam.
- Psychiatry Consult with pending admit to Acute Inpatient Psychiatric Facility and provide appropriate referral.
- Perform Urinary Drug Screen and give appropriate referral.
- Define Severity of psychosis through PANSS^6^
- Perform standardized needs assessment with Family Development Matrix^2^
- Coordinate care with social
- services based on needs assessment.
- Patient meets Homeless Vulnerability Index, prioritize needs for housing.^7^

Cluster 5-Substance Use Disorder Cluster

- Proceed with standard medical exam.
- Psychiatry Consult with pending admit to Acute Inpatient Psychiatric Facility and provide appropriate referral.
- Perform Urinary Drug Screen and give appropriate referral
- Perform standardized needs assessment with Family Development Matrix^2^
- Coordinate care with social services based on needs assessment.

Cluster 6-Hypertension Cluster

- Proceed with standard medical exam.
- Perform Urinary Drug Screen and give appropriate referral
- Perform standardized needs assessment with Family Development Matrix^2^
- Coordinate care with social services based on needs assessment.

Cluster 8-Infection Cluster

- Proceed with standard medical exam.
- Perform Urinary Drug Screen and give appropriate referral.
- Define HIV/STD/Hepatitis Infection status through HIV/STD/Hepatitis Risk Assessment Questionnaire^8^ and provide referral to PCP.
- Symptoms of Tuberculosis (productive cough >3 weeks, hemoptysis, unexplained weight loss, fever/chills/night sweats, unexplained fatigue, chest pain and provide referral to PCP
- Perform standardized needs assessment with Family Development Matrix^2^
- Coordinate care with social services based on needs assessment.

Cluster 9-Injury Group

- Proceed with standard medical exam.
- Perform Urinary Drug Screen and provide appropriate referral.
- Perform standardized needs assessment with Family Development Matrix^2^
- Coordinate care with social services based on needs assessment.

Cluster 7-COPD Cluster

- Proceed with standard medical exam.
- Perform Urinary Drug Screen and give appropriate referral.
- Perform standardized needs assessment with Family Development Matrix^2^
- Coordinate care with social services based on needs assessment.

**Citations**

1. McNeely, J., Wu, L. T., Subramaniam, G., Sharma, G., Cathers, L. A., Svikis, D., ... & Schwartz, R. P. (2016). Performance of the tobacco, alcohol, prescription medication, and other substance use (TAPS) tool for substance use screening in primary care patients. *Annals of internal medicine*, *165*(10), 690-699.
2. Running ross step-by-step. (n.d.). Retrieved February 26, 2021, from https://www.hudexchange.info/programs/ross/guide/working-with-residents/how-should-service-coordinators-approach-needs-assessments-and-goal-setting-with-residents/what-types-of-needs-assessment-tools-should-service-coordinators-use/
3. Horowitz, L. M., Snyder, D. J., Boudreaux, E. D., He, J. P., Harrington, C. J., Cai, J., ... & Pao, M. (2020). Validation of the Ask Suicide-Screening Questions for adult medical inpatients: a brief tool for all ages. *Psychosomatics*, *61*(6), 713-722.
4. Martinez, F. J., Raczek, A. E., Seifer, F. D., Conoscenti, C. S., Curtice, T. G., D'Eletto, T., ... & Phillips, A. L. (2008). Development and initial validation of a self-scored COPD Population Screener Questionnaire (COPD-PS). *COPD: Journal of Chronic Obstructive Pulmonary Disease*, *5*(2), 85-95.
5. Posner, K., Brown, G. K., Stanley, B., et al. (2011). The Columbia-Suicide Severity Rating Scale: initial validity and internal consistency findings from three multisite studies with adolescents and adults. American Journal of Psychiatry, 168(12), 1266–1277.
6. Bull, P. (2017). Rating scales and safety measurements in bipolar disorder and schizophrenia–a reference guide. *Psychopharmacology Bulletin*, *47*(3), 77-109.
7. Babcoc Brown, M., Cummings, C., Lyons, J., Carrión, A., & Watson, D. P. (2018). Reliability and validity of the Vulnerability Index-Service Prioritization Decision Assistance Tool (VI-SPDAT) in real-world implementation. *Journal of Social Distress and the Homeless*, *27*(2), 110-117.
8. k, M., & Pretzel, E. (2016). Risk Assessment Tool: HIV/STD/Hepatitis-Minnesota Dept. of Health. MN Department of Health. https://www.health.state.mn.us/diseases/stds/riskassess/riskassess.pdf.
